# Assessment of Anxiety, Depression and Work-Related Strain Levels of Healthcare Professionals Working in Operating Rooms and Intensive Care Units During the COVID-19 Outbreak

**DOI:** 10.5152/TJAR.2022.211139

**Published:** 2022-04-01

**Authors:** Hale Kefeli Çelik, Zahide Doğanay, Sevgi Canbaz, Gözde Yontar

**Affiliations:** 1 Department of Anesthesiology and Reanimation, Health Sciences University, Samsun Training and Research Hospital, Samsun, Turkey; 2 Department of Public Health, İstanbul University Faculty of Medicine, İstanbul, Turkey; 3 Department of Psychiatry, Health Sciences University, Samsun Training and Research Hospital, Samsun, Turkey

**Keywords:** Anxiety, COVID-19, depression, healthcare professional, intensive care, operating room, work-related strain

## Abstract

**Objective:**

The purpose of the study was to determine the levels of work-related strain, anxiety, and depression in health professionals working in operating rooms and intensive care units who deal with the diagnosis, treatment, and care of coronavirus disease 19 patients.

**Methods:**

The population of the study consisted of 320 healthcare professionals working in the operating room and intensive care units. After providing detailed information about the study to the participants, a questionnaire consisting of 21 questions including sociodemographic information and working life characteristics, 14 questions from the Hospital Anxiety and Depression Scale, and 18 questions from the Work-Related Strain Inventory (WRSI) were administered under supervision.

**Results:**

In total, 58.8% of the participants were working in intensive care units, and 41.2% of the participants were in the operating room. The scores obtained from Work-Related Strain Inventory were found to be statistically significantly high in those who wanted to choose a different profession, those who were on duty during the coronavirus disease 19 pandemic process, those whose spouses followed coronavirus disease 19 patients during the pandemic, those who encountered a suspicious situation and had a coronavirus disease 19 test, those who had difficulty in accessing personal protective equipment, and those who thought that their lives were in danger during the pandemic process. Participants with anxiety risk according to Hospital Anxiety and Depression Scale-Anxiety subscale and depression risk according to Hospital Anxiety and Depression Scale-Depression subscale were found to be 153 (47.8%) and 300 (93.8%), respectively.

**Conclusions:**

It was found that Work-Related Strain Inventory and anxiety-depression rates were significantly high in both the operating room and intensive care unit workers who actively provided healthcare services to patients diagnosed with coronavirus disease 19.

**Main Points**

- The coronavirus disease 19 (COVID-19) outbreak is an important issue for frontline healthcare professionals (HPs) who provide healthcare to patients and are in direct contact with them.
- Healthcare professionals in ICUs and operating rooms fighting against an unknown enemy, must use personal protective equipment and be extremely careful.
- In these cases, algorithms and guidelines published by the specialty associations and the Ministry of Health Scientific Committee should be utilized.
- During the COVID-19 outbreak, as in other studies, we found a high rate of work-related strain, anxiety, and depression in our HPs. Independent risk factors for anxiety and depression were tobacco use and seeing one’s life in danger during the pandemic process.

## Introduction

The new coronavirus disease 19 (COVID-19), also known as the new coronavirus pneumonia, first appeared in Wuhan province of China at the beginning of December and spread almost all over the world within 2 months, causing a pandemic. Coronavirus disease 19 is caused by a virus called severe acute respiratory syndrome coronavirus 2 (SARS-CoV-2). Even though the overall mortality rate of the disease is around 2%, in some populations, this rate may increase by 50% and in those who have contracted COVID-19 disease, 80% have mild disease and 20% require hospitalization, and some of these patients need to be followed up in the intensive care unit (ICU).^1^

Work-related strain (WRS) is more common in people in professions that have intense and continuous relationships. Reasons such as workload, taking responsibility for the patient, caring for serious and terminally ill patients, and having to provide emotional support to patients and their relatives when necessary cause work-related stress and tension in healthcare professionals (HPs). WRS causes psychological effects such as depression, anxiety, and helplessness in the person, and physiological effects such as headache, muscle tension, and insomnia. Today, it is known that WRS gradually increases among the HPs working in ICUs and this causes anxiety and depression in the employees over time. It is predicted that these complaints are more common in HPs who work in the COVID-19 pandemic and may cause serious psychological problems in healthcare workers.^2,3^

The study aimed to determine the levels of WRS, anxiety, and depression in HPs working in operating rooms and ICUs who deal with the diagnosis, treatment, and care of COVID-19 patients.

## Methods

This cross-sectional study was conducted between June 5 and 15, 2020 with the permission of the Ministry of Health General Directorate of Health Services dated May 27, 2020, and the ethics committee of Health Sciences University Samsun Training and Research Hospital dated June 05, 2020, and numbered 2020/08. The study adhered to the Declaration of Helsinki.^4^ The population of the study consisted of 350 HPs working in the operating room and ICUs of the hospital, which is the primary pandemic hospital in the region. After giving detailed information about the study to the participants, the questionnaire was applied, and 320 (91.4%) participants completed the questionnaire. A questionnaire consisting of 21 questions including sociodemographic information, working life characteristics, changes in the working environment due to the COVID-19 pandemic, 14 questions from the Hospital Anxiety and Depression Scale (HADS), and 18 questions from the Work-Related Strain Inventory (WRSI) was applied to the participants under supervision.

The exclusion criteria from the study were as follows: HPs not working in ICUs and operating rooms, HPs who did not voluntarily consent to participate, HPs on administrative leave during the COVID-19 pandemic.

### Scales Used in the Study

*Hospital Anxiety and Depression Scale* was developed by Zigmond et al^5^ and its validity and reliability study was performed in 1983. The purpose of the scale is not to make a diagnosis but to determine the risk group by screening anxiety and depression. This scale, which is a self-report scale, includes 14 items, 7 of which are about depression (even-numbered questions) and 7 of which are about anxiety (odd-numbered questions). It has a 4-point Likert-type response system and the responses are scored between 0 and 3. The scoring of each item on the scale is different. Items 1, 3, 5, 6, 8, 10, 11, and 13 indicate decreasing levels of severity and are scored as 3, 2, 1, and 0. Items 2, 4, 7, 9, 12, and 14 are scored as 0, 1, 2, and 3. While the scores of items 1, 3, 5, 7, 9, 11, and 13 are summed up for the anxiety subscale, the scores of items 2, 4, 6, 8, 10, 12, and 14 are summed up for the depression subscale. The lowest score that patients can get from both subscales is 0 and the highest score is 21. The cut-off points of the Turkish version of HADS were determined as 10 for the anxiety subscale (HADS-A) and 7 for the depression subscale (HADS-D). The validity and reliability of the Turkish form of the scale were carried out by Aydemir et al^6^ and the Cronbach’s alpha coefficient for the anxiety subscale was found to be 0.8525 and 0.7784 for the depression subscale, and it was determined that the scale could be used reliably. 

*Work-Related Strain Inventory* is a 4-point Likert-type self-report scale with 18 items developed to determine work-related stress in HPs. It is scored between 1 and 4 points. Items 2, 3, 8, 9, 11, and 15 are reversely scored. The lowest score to be obtained from the scale is 18, and the highest score is 72. The scale does not have a cut-off value, and the level of WRS increases in direct proportion to the score obtained from the scale. Work-Related Strain Inventory was developed by Revicki et al^7^ in 1991. The adaptation of the scale to Turkish and its validity–reliability study were conducted in 1998 by Aslan et al.^8^

### Statistical Analysis

Statistical Package for the Social Sciences version 21 (IBM Corp.; Armonk, NY, USA) was used to evaluate the data. When the age, duration of employment, and scale scores obtained as a result of the study were evaluated using the Kolmogorov–Smirnov test in terms of suitability for a normal distribution, it was found that they did not comply with a normal distribution. These data were given as median (minimum-maximum), and the Mann–Whitney *U* test was used to compare binary groups in the evaluation of the data. The Kruskal–Wallis test was used in the comparison of more than 2 subgroups, and the significant parameters were compared with the Bonferroni-corrected Mann–Whitney *U* test. The statistical significance level was taken as *P* < .05, and after Bonferroni correction, it was taken as *P* < .01. 

## Results

The median age of the 320 HPs participating in the study was 38.0 (19.0-55.0) years, the median duration of employment in the profession was 13 (1-35) years, and 66.6% (n = 213) of them were female. In terms of job titles, 52.2% of the participants were nurses and 15.9% were assistant personnel. Sociodemographic characteristics and professional information of the participants are summarized in Table 1. 

In total, 259 of the participants (80.9%) stated that they were on duty during the pandemic and 96 (30.0%) of them used tobacco products. Again, 200 of the participants (62.5%) stated that they would choose a different profession if they had a choice at the time of the study. During the pandemic, 81.6% of the participants provided services to COVID-19 patients; the spouses of 21.6% of them were determined to provide health services to COVID-19 patients; 57.2% of the participants continued to stay in the same house with their family during the pandemic process; 78.1% stated that their lives were in danger during this period; 27.2% of the participants stated that they had difficulty in accessing personal protective equipment (PPE) during the pandemic period. During the pandemic period, 27 (8.4%) of the participants had a COVID-19 polymerase chain reaction (PCR) test due to contact history and/or suspected disease symptoms, and all of them were reported as negative. The difficulties experienced by the participants during the pandemic are presented in Table 2.

Work-Related Strain Inventory score was found to be statistically significantly lower in divorced workers and specialist physicians (*P* < .05). However, the participants whose score was statistically significantly higher (*P* < .05) can be listed as follows: those who wanted to choose a different profession, those who were on duty during the COVID-19 process, those whose spouses followed up COVID-19 patients during the pandemic, those who encountered a suspicious situation and had a COVID-19 PCR test, those who had difficulty in accessing PPE, those who thought their lives were in danger during the pandemic process, those who felt helpless, and those whose sleep and eating patterns deteriorated compared to pre-pandemic period (Table 3).

Participants with anxiety risk according to HADS-A and depression risk according to HADS-D were 153 (47.8%) and 300 (93.8%), respectively. While there was no statistically significant difference in any of the comparisons in the HADS-A scale, a statistically significant difference (*P* < .05) was found on the HADS-D scale according to the use of tobacco products and the state of seeing their lives in danger during the pandemic process. The comparison of the scores obtained by the participants from the scales according to some characteristics is presented in Table 3.

## Discussion

The World Health Organization declared COVID-19 disease as a pandemic on March 11, 2020, and the first cases were seen on the same dates in Turkey. In the following period, the number of patients diagnosed with COVID-19 in our country gradually increased and began to be seen in every province. With the decision of the Turkish Ministry of Health, all hospitals in the provinces were accepted as pandemic hospitals, and patients were admitted to both normal wards and ICUs. Turkish Ministry of Health published a new guideline almost every week and made recommendations in both patient diagnosis, follow-up, and treatment, thanks to the scientific committee that closely followed the developments in all countries. Also, HPs who were actively in contact with patients diagnosed with COVID-19 were trained on protection methods, and efforts were made to find solutions for the provision of PPE in hospitals. In the same period, the Turkish Society of Anaesthesiology and Reanimation and the Turkish Society of Intensive Care published up-to-date guidelines and algorithms on the COVID-19 disease, as did all specialist associations. In these guidelines, intervention, treatment methods, and personal protection methods for suspected or confirmed COVID-19 patients were described for anaesthesiologists, intensive care specialists, operating room, and ICU personnel.^9,10^

Healthcare professionals work in environments with more WRS due to many reasons such as intensive workload, providing care for serious and terminally ill patients, and providing emotional support to patients and their relatives. Work-related strain and stress are concepts that are often used interchangeably. A WRS occurs as a result of the interaction of the stressful situations in the work environment, the conditions of the work environment, and the characteristics of the individual. Stress is an external burden on different psychological, social, or biological systems, while tension is defined as the negative effects caused by stress on these systems. When the duration and frequency of WRS exceed the person’s capacity to cope, problems begin to emerge, and as a result, psychological problems such as anxiety and depression are observed.^11,12^ Anxiety and depression are common mental health problems that affect the quality of life of people and lead to production losses in workplaces. The lifetime prevalence for anxiety disorders in adults is reported as 33.7% and for depression as 10%-15%.^13^

Healthcare professionals are at high risk in terms of their physical and mental health in times of epidemics and pandemics. Causes of mental health decline include extreme workload, unpreparedness, and emotional distress (fear of infection, family concerns, etc.). Epidemiological studies conducted to date have shown that SARS and the Middle East Respiratory Syndrome Coronavirus (MERS-CoV) caused a high rate of psychological morbidity in HPs in hospitals. Compared to previous outbreaks, the COVID-19 outbreak differs due to the high contagiousness of the virus, lack of knowledge of treatment methods, lack of sufficient information about the course, and consequences of the infection. Especially, in the study conducted by Lü et al^13^ which investigated the psychological effects on HPs during SARS, this rate was found to be 39.3%, while Xiao et al^14^ reported that the rate was 55.1% in the COVID-19 pandemic. In this study conducted in our hospital, which is the primary pandemic hospital in our city, 81.6% of the participants provided health services to COVID-19 patients and 58.8% were working in ICUs. In our study, the anxiety rate was found to be 47.8%, and the depression rate was as high as 93.8% in the HPs working in these departments. 

Karanikola et al^15^ reported that nurses working in ICUs faced a higher psychological burden compared to other units and the general population, with an anxiety prevalence ranging from 10.2% to 32% and depression symptoms at rates varying between 11% and 31%. In studies conducted during the pandemic period, when indicators such as fear of infection and stress in the workplace associated with COVID-19 were evaluated, it was shown that the rate of psychological tension and burnout was high and that the COVID-19 epidemic was an emotionally and physically more stressful situation. Zerbini et al^16^ reported that those who were in constant and direct contact with COVID-19 patients, especially nurses, were at higher risk in terms of psychological burden. Again, in a cross-sectional study, Wang et al^17^ evaluated the levels of depression, stress, and anxiety at the onset of the COVID-19 outbreak and reported that 53.8% of the participants showed evidence of severe psychological effects of the outbreak. Totally 52.2% of the participants in our study were nurses, and 66.6% were female. While there was no difference in WRSI scores in terms of gender, WRSI scores were higher in nurses and research assistants than others, which should be a natural situation for these HPs who were in direct contact with the patients. 

According to the data of the Chinese Center for Disease Control and Prevention, as of February 11, 2020, it was estimated that 3000 HPs were infected with COVID-19 and that 1716 of them tested positive for COVID-19.^18^ Wu et al^19^ reported that during the epidemic period of COVID-19, healthcare personnel, especially in the Wuhan area, had higher levels of psychological stress than university students and showed an “exposure effect” to the disease. They also reported that HPs working in the Wuhan area had higher feelings that the crisis was approaching compared to other regions and were more confident as regards defeating the COVID-19 outbreak.

Again in a study conducted in Wuhan, where COVID-19 disease occurred, Xiao et al^14^ stated that 30% of the participants worked in ICUs and departments dealing with respiratory diseases, that 65% had contact with patients with confirmed or suspected COVID-19 disease, and that 60.8% could not have access to PPE. In the light of all these data, anxiety was detected in 54.1% of the participants, and depression was found in 57.3% of the participants, and gender, profession, access to PPE, and contact history were reported as risk factors.^14^ During the pandemic period, 42.8% of the participants in our study did not stay in the same house with their families and 78.1% stated that their lives were in danger. Especially in participants who had difficulties in accessing PPE, the WRSI score was significantly higher. Again, it was found that 53.4% of the participants felt helpless in the face of this disease and that both their sleep and eating patterns were significantly impaired compared to the pre-pandemic period.

In a recent study conducted among HPs in our country, Şahin et al^20^ found that 87.1% of the participants were afraid of being infected with COVID-19 and therefore washed their hands 15 times a day on average (min: 2, max: 95) and that burnout levels were higher in those who worked especially in pandemic outpatient clinics and ICUs. Besides, as stated in the literature, in this study, anxiety and burnout rates were observed to be higher in female HPs,^14,21^ and it was also observed that the symptoms of fear, anxiety, and burnout were higher in HPs who lived with their family during the pandemic process, especially those who have children and a family member of above 65 years of age at home.^20^ In our study, when the participants were asked if they had a chance to “choose a different profession” during the pandemic period, the answer “we would choose” was obtained at a statistically significant rate. The rates of anxiety and depression were found to be significantly different, especially in personnel using tobacco products. This may have stemmed from the fact that smoking was mentioned as an important risk factor in COVID-19 infection that causes pneumonia, which may have had a negative effect on the personnel. 

Psychological factors play a vital role during any pandemic. For example, the measures are taken, and the attitudes of a population toward social distancing have critical effects on the spread of infection. Besides, psychological factors can lead to increased psychological distress related to how people deal with the threat of infection, the fear of losing loved ones, and the grief of actually losing loved ones.^22^ Tajvar et al^23^ reported in their study with intensive care nurses that the rate of somatic symptoms, anxiety, depression, and a mental disorder was higher in nurses who were married. In our study, when compared to divorced individuals, the high rates of WRSI scores in married and single healthcare personnel reveal both the risk of married personnel for infecting relatives and the tension experienced by single and young inexperienced personnel. In our study, the low number of participants who had the COVID-19 PCR test (8.4%) and all of the tests performed being a negative show that the personnel complied with the recommended protection and intervention methods. 

In Turkey and other countries, the number of patients diagnosed with COVID-19 is increasing every passing day and these cases can prove fatal. While some of these patients are treated in normal service rooms, some of them, especially patients diagnosed with pneumonia and having respiratory difficulties, are treated in ICUs. In some cases, these patients may need to be operated and even emergency intervention is required. In this context, anaesthesiologists, intensive care specialists, and assistant staff should have knowledge and training on the intervention with these patients. Healthcare professionals in ICUs and operating rooms fighting against an unknown enemy must be extremely careful. In these cases, algorithms and guidelines published by the specialty associations and the Ministry of Health Scientific Committee should be utilized.

As a result, the COVID-19 outbreak is an important issue for frontline HPs who provide healthcare to patients and are in direct contact with them, as well as for public health. During the epidemic period, stress, anxiety, and other negative emotions arise in the country and spread rapidly among the members of the society, dragging the whole country into a psychological crisis. Healthcare professionals are doing great work under psychological pressure that affects their emotional state, which leads to the development of psychological stress. Healthcare professionals serving at the forefront of the epidemic for long periods show stronger psychological stress to cope with more negative emotional distress. During the COVID-19 outbreak, as in other studies, we found a high rate of WRS, anxiety, and depression in our HPs. Independent risk factors for anxiety and depression were tobacco use and seeing one’s life in danger during the pandemic process. It shows that during the epidemic, it is important that HPs have access to protective equipment support and that staff whose spouses also serve patients diagnosed with COVID-19 disease should be given primary attention and psychological support. Measures to be taken in the light of all these results will ensure that a crisis that will emerge will remain at a minimal level.

The limitations of this study are that we had not measured the psychological effects on our personnel working in the ICUs and operating rooms before the epidemic. Therefore, the effects caused by non-epidemic causes cannot be excluded. In addition, it is not correct to analyze the continuous psychological effect with this cross-sectional study.

### Declaration of Interest:

The authors have no conflict of interest to declare.

## Figures and Tables

**Table 1.** Demographic and Occupational Characteristics of the Participants

Characteristics of the Participants	n = 320	%
**Gender**		
Female	213	66.6
Male	107	33.4
**Marital status**		
Married	223	69.7
Single	72	22.5
Divorced	25	7.8
**Profession**		
Nurse	167	52.2
Personnel	51	15.9
Anaesthetist technician	56	17.5
Anaesthetist	29	9.1
Research assistant	11	3.4
Surgical technician	6	1.9
**Workplace**		
Intensive care unit	188	58.8
Operating room	132	41.2
**Have a child**		
Yes	230	71.9
No	90	28.1

**Table 2.** Difficulties Experienced by Participants During the Pandemic Period

Difficulties Experienced	n = 320	%
Those whose spouses also followed up COVID-19 patients during the pandemic	69	21.6
Those staying in the same house with their family during the pandemic	183	57.2
Those providing healthcare services to COVID-19 patients	261	81.6
Those having COVID-19 test*	27	8.4
Those experiencing difficulty in accessing personal protective equipment	87	27.2
Those seeing their lives in danger during the pandemic process	250	78.1
Those feeling helpless during the pandemic	171	53.4
Those whose sleep patterns deteriorated in comparison to the pre-pandemic period	237	74.1
Those whose eating pattern deteriorated in comparison to the pre-pandemic period	203	63.4
Those who lost their relative/acquaintance due to COVID-19	26	8.1

*There were no healthcare professionals with positive test results during the study period.

**Table 3.** Evaluation of the Participants According to WRSI and HADS Scores

	Work-Related Strain Inventory Median (Min-Max)	HADS-A*** (n)	HADS-D*** (n)
Gender*			
Female	47 (29-72)	95	203
Male	49 (33-72)	58	97
* P*	.162	.105	.105
Marital status**			
Married^1^	47.5 (33-72)	109	210
Single^2^	49 (38-72)	37	67
Divorced^3^	44 (29-57)	7	23
* P*	**.022**	.141	.957
Profession**			
Anaesthetist	43 (29-60)	13	28
Research assistant	49 (39-57)	3	11
Nurse	48 (33-72)	84	155
Anaesthetist technician	47.5 (29-59)	3	54
Surgery technician	49.5 (47-60)	1	5
Personnel	48 (40-72)	19	46
* P*	**.002**	.577	.138
Workplace*			
Intensive care unit	47 (29-72)	85	174
Operating room	49 (29-64)	68	126
* P*	.272	.500	.523
Have a child*			
Yes	49 (37-72)	106	215
No	48 (29-64)	37	64
* P*	.847	.188	.537
Smoke*			
Yes	48 (29-72)	47	86
No	47.5 (29-72)	106	214
* P*	.354	.788	**.044**
Wanted to choose a different profession during the pandemic period*			
Yes	50 (33-64)	61	115
No	47 (29-62)	92	185
* P*	**.005**	.402	.233
On duty during the COVID-19 period*			
Yes	51 (33-64)	124	243
No	47 (29-72)	29	57
* P*	<.001	.630	.958
Spouses also followed up COVID-19 patients during the pandemic period*			
Yes	48 (29-64)	32	68
No	46 (33-72)	80	147
* P*	**.012**	.615	.053
Staying in the same house with their family during the pandemic period*			
Yes	48 (33-64)	90	170
No	48.5 (29-59)	16	46
* P*	.355	.050	.462
Care to COVID-19 patient*			
Yes	47 (29-72)	126	247
No	49 (36-72)	27	53
* P*	.084	.727	.168
Having COVID-19 test*			
Yes	48 (29-72)	16	26
No	45.5 (29-57)	137	274
* P*	**.042**	.301	.838
Experiencing difficulty in accessing personal protective equipment*			
Yes	49 (33-72)	43	81
No	45 (29-60)	110	219
* P*	<.001	.724	.770
Seeing their lives in danger during the pandemic process*			
Yes	50.5 (33-72)	123	238
No	47 (29-72)	30	61
* P*	**.003**	.395	**.035**
Feeling helpless during the pandemic*			
Yes	50 (33-72)	82	160
No	46 (29-61)	71	140
* P*	<.001	.957	.885
Sleep patterns deteriorated in comparison to the pre-pandemic period*			
Yes	52 (36-64)	107	223
No	46 (29-72)	46	77
* P*	<.001	.269	.053
Eating pattern deteriorated in comparison to the pre-pandemic period*			
Yes	52 (36-64)	92	191
No	46 (29-72)	59	104
* P*	<.001	.638	.399
Lost their relative/acquaintance due to COVID-19*			
Yes	48 (29-72)	17	25
No	46.5 (36-57)	136	275
* P*	.380	.073	.810
*Mann–Whitney *U* test; **Mann–Whitney *U* test with Bonferonni correction after Kruskal–Wallis test; ***Chi-square test 1.2 *P* = .430; 1.3 *P* = .016; 2.3 *P* = .004.HADS-A, Hospital Anxiety and Depression Scale-Anxiety Subscale; HADS-D, Hospital Anxiety and Depression Scale-Depression Subscale; WRSI, Work-Related Strain Inventory.			

## References

[1] Sun N Wei L Shi S et al. A qualitative study on the psychological experience of caregivers of COVID-19 patients. Am J Infect Control. 2020;48(6):592 598. 10.1016/j.ajic.2020.03.018) 32334904 PMC7141468

[2] Tokuç B Turunç Y Ekuklu G . Edirne’de Ambulans Çalışanlarının Anksiyete, Depresyon ve Işe Bağlı Gerginlik Düzeyleri. TTB Mesleki Sağlık Güvenlik Derg. 2011;11:39 44.

[3] Tan BYQ Chew NWS Lee GKH et al. Psychological impact of the COVID-19 pandemic on health care workers in Singapore. Ann Intern Med. 2020;173(4):317 320. 10.7326/M20-1083) 32251513 PMC7143149

[4] Williams JR The Declaration of Helsinki and public health. Bull World Health Organ. 2008;86(8):650 652. 10.2471/blt.08.050955) 18797627 PMC2649471

[5] Zigmond AS Snaith RP . The hospital anxiety and depression scale. Acta Psychiatr Scand. 1983;67(6):361 370. 10.1111/j.1600-0447.1983.tb09716.x) 6880820

[6] Aydemir Ö Güvenir T Küey L Kültür S . Hastane Anksiyete ve Depresyon Ölçeği Türkçe Formunun Geerlilik ve Güvenirlik Çalışması. Türk Psikiyatr Derg. 1997;8(4):280 287.

[7] Revicki DA May HJ Whitley TW . Reliability and validity of the work-related strain inventory among health professionals. Behav Med. 1991;17(3):111 120. 10.1080/08964289.1991.9937554) 1932844

[8] Aslan H Alparslan N Aslan O Kesepera C Ünal M . İşe Bağlı Gerginlik Ölçeğinin Sağlık Alanında Çalışanlarda Geçerlik ve Güvenirliği. Düşünen Adam Derg. 1998;11(2):4 8.

[9] Türk Anesteziyoloji ve Reanimasyon Derneği. Available at: https://www.tard.org.tr/haberler/1529.

[10] Türk Yoğun Bakım Derneği. Available at: https://www.yogunbakim.org.tr/haberler/7661/COVID-19-Yogun-Bakim-unitesi-Triaj-Algoritmasi#.

[11] Yürür S Keser A . The mediating role of emotional exhaustion on job related strain and job satisfaction relationship. Ank Univ Sağlık Bilimleri Fak Derg. 2010;65(4):165 194.

[12] Kılıç Ü Yön B Şişman NY . The relationship between work-related stress and the risk of anxiety and depression of emergency station personnel. Turk J Public Health. 2019;17(2):143 152.

[13] Lü SH Tian BC Yang TZ Chen DW Chi YH . Perceived stress in general public during prevalence of severe acute respiratory syndrome and its impact on health behavior. Zhonghua Yu Fang Yi Xue Za Zhi. 2010;44(2):128 133. 20388333

[14] Xiao X Zhu X Fu S Hu Y Li X Xiao J . Psychological impact of healthcare workers in China during COVID-19 pneumonia epidemic: a multi-center cross-sectional survey investigation. J Affect Disord. 2020;274:405 410. 10.1016/j.jad.2020.05.081) 32663970 PMC7236675

[15] Karanikola M Giannakopoulou M Mpouzika M Kaite CP Tsiaousis GZ Papathanassoglou ED . Dysfunctional psychological responses among Intensive Care Unit nurses: a systematic review of the literature. Rev Esc Enferm USP. 2015;49(5):847 857. 10.1590/S0080-623420150000500020) 26516757

[16] Zerbini G Ebigbo A Reicherts P Kunz M Messman H . Psychosocial burden of healthcare professionals in times of COVID-19 – a survey conducted at the University Hospital Augsburg. Ger Med Sci. 2020;18:Doc05. 10.3205/000281) PMC7314868 32595421

[17] Wang C Pan R Wan X et al. Immediate psychological responses and associated factors during the initial stage of the 2019 coronavirus disease (COVID-19) epidemic among the general population in China. Int J Environ Res Public Health. 2020;17(5):1729. 10.3390/ijerph17051729) PMC7084952 32155789

[18] China Disease Control Report: more than 3,000 medical staff infected with new crown virus [EB / OL]; 2020. Available at: http://hb.sina.com.cn/news/n/2020-02-18/detail-iimxyqvz3722095.shtml. Accessed February 18, 2020.

[19] Wu W Zhang Y Wang P et al. Psychological stress of medical staffs during outbreak of COVID-19 and adjustment strategy. J Med Virol. 2020;92(10):1962 1970. 10.1002/jmv.25914) 32314806 PMC7264502

[20] Sahin T Aslaner H Eker OO Gokcek MB Dogan M . Effect of COVID-19 Pandemic on Anxiety and Burnout Levels in Emergency Healthcare Workers: a Questionnaire Study. Research Square; 2020. 10.21203/rs.3.rs-32073/v1)

[21] McMurray JE Linzer M Konrad TR Douglas J Shugerman R Nelson K . The work lives of women physicians results from the physician work life study. The SGIM Career Satisfaction Study Group. J Gen Intern Med. 2000;15(6):372 380. 10.1046/j.1525-1497.2000.9908009.x) 10886471 PMC1495474

[22] Morgul E Bener A Atak M et al. COVID-19 pandemic and psychological fatigue in Turkey. Int J Soc Psychiatry. 2021;67(2):128 135. 10.1177/0020764020941889) 32650681 PMC7355205

[23] Tajvar A Saraji GN Ghanbarnejad A Omidi L Hosseini SSS Abadi ASS . Occupational stress and mental health among nurses in a medical intensive care unit of a general hospital in Bandar Abbas in 2013. Electron Phys. 2015;7(3):1108 1113. 10.14661/2015.1108-1113) PMC4574696 26388976

